# Estimate of Dietary Total Antioxidant Capacity of Pregnant Women and Associated Factors

**DOI:** 10.1055/s-0041-1741454

**Published:** 2022-02-25

**Authors:** Mariana Rinaldi Carvalho, Lívia Castro Crivellenti, Daniela Saes Sartorelli

**Affiliations:** 1 Department of Social Medicine, Public Health Program, Faculdade de Medicina de Ribeirão Preto, Universidade de São Paulo, Ribeirão Preto, SP, Brazil; 2 Department of Social Medicine, Faculdade de Medicina de Ribeirão Preto, Universidade de São Paulo, Ribeirão Preto, SP, Brazil

**Keywords:** total antioxidant capacity, pregnant women, diet, antioxidants, capacidade antioxidante total, gestantes, dieta, antioxidantes

## Abstract

**Objective** To investigate the dietary total antioxidant capacity (DTAC) of pregnant women, and associated factors.

**Methods** Cross-sectional study conducted with 785 pregnant adult women attended in primary health care centers of Ribeirão Preto, state of São Paulo, Brazil. Two 24-hour dietary recalls were obtained, and the usual intake was estimated through the Multiple Source Method. The DTAC was estimated using the ferric reducing antioxidant power assay. The relationship between the higher DTAC estimate (≥ median of 4.3 mmol/day) and associated factors was investigated using adjusted logistic models with backward selection.

**Results** In total, 25% of the pregnant women were classified as overweight, and 32% as obese. The median (P25, P75) DTAC was 4.3 (3.3–5.6) mmol/day. Through adjusted logistic regression models with backward selection, a higher chance of DTAC estimates above the median among pregnant women aged ≥ 35 years old (2.01 [1.24–3.27]) was verified when compared with younger pregnant women. Women with prepregnancy overweight (0.63 [0.45–0.89]) and obesity (0.59 [0.40–0.88]) presented a lower chance of DTAC estimates above the median when compared with eutrophic pregnant women. A higher DTAC estimate was positively associated with the use of dietary supplements (1.39 [1.03–1.88]), and negatively associated with total dietary energy (0.59 [0.42–0.85]).

**Conclusion** The DTAC estimate over the median was associated with greater age, adequate body weight, use of dietary supplements, and lower energy intake.

## Resumo

**Objetivo** Investigar a capacidade antioxidante total da dieta (CATd) de gestantes e os fatores associados.

**Métodos** Estudo transversal conduzido entre 785 gestantes adultas em acompanhamento de pré-natal em Unidades Básicas de Saúde de Ribeirão Preto, São Paulo, Brasil. Para a estimativa da dieta usual, 2 inquéritos recordatórios de 24 horas foram obtidos e ajustados empregando-se o Multiple Source Method. A CATd foi estimada com base no ensaio de potência de redução de ferro. Para investigar a relação entre a maior estimativa de CATd (≥ mediana de 4,3 mmol/dia) e os fatores associados, foram empregados modelos de regressão logística ajustados em função backward *.*

**Resultados** No total, 25% das gestantes foram classificadas com sobrepeso e 32% com obesidade. A mediana (P25; P75) da CATd foi de 4,3 (3,3–5,6) mmol/dia. Em modelos de regressão logística ajustados em função backward *,* verificou-se maior chance da estimativa da CATd acima da mediana entre gestantes com idade ≥ 35 anos (2,01 [1,24–3,27]), quando comparadas às gestantes de menor idade. Mulheres com sobrepeso (0,63 [0,45–0,89]) e obesidade (0,59 [0,40; 0,88]) no período pré-gestacional apresentaram menor chance da estimativa da CATd acima da mediana, quando comparadas às eutróficas. A estimativa da CATd foi positivamente associada ao uso de suplementos dietéticos (1,39 [1,03–1,88]) e negativamente associada à energia total da dieta (0,59 [0,42–0,85]).

**Conclusão** A estimativa da CATd acima da mediana foi positivamente associada à maior idade, eutrofia, ao uso de suplementos dietéticos e à menor ingestão energética.

## Introduction

Maternal nutrition during pregnancy has a great influence on birth outcomes and on the development of chronic diseases in adulthood. 1 Evidence suggests that adherence to healthy eating patterns composed of foods that are sources of antioxidants, such as fruits, legumes, and vegetables (FLVs), have a protective effect during pregnancy in relation to prematurity and low birth weight (LBW), promoting adequate childhood growth and development. 2

Numerous vitamins and minerals with antioxidant properties are used to explain the pathway that connects maternal healthy eating behavior with fetal growth. Increased maternal intake of vitamin D supplements reduces the risk of small for gestational age (SGA) infants. 3 Furthermore, the adequate intake of zinc, magnesium, calcium, and vitamin D supplements is associated with a reduction in oxidative stress in pregnant women with gestational diabetes mellitus (GDM). 4

Higher levels of reactive oxygen species are physiologically checked during pregnancy and may in fact be important for its organogenesis. 5 However, excessive production of free radicals associated with a low antioxidant defense can negatively impact the development of the placenta and, consequently, the health of the newborn. 5 To inhibit and/or reduce the damage caused by the action of free radicals, the human body has enzymatic and nonenzymatic antioxidant defense systems. 6

The enzymatic defense system includes the enzymes superoxide dismutase, peroxidase, catalase, and glutathione-peroxidase. The activity of these enzymes depends on the participation of nonenzymatic cofactors, the diet being the main contributing factor for the regulation of the serum antioxidant status. 7

Dietary antioxidants evaluated in isolation may not reflect the total antioxidant power of the diet, as this does not consider the additive or synergistic effects of the interaction between them. Therefore, the dietary total antioxidant capacity (DTAC) has been used to investigate the potential antioxidant effects of foods present in the diet, considering the synergy between them. 8 The DTAC is recognized as a potential marker of the quality of the usual diet and is positively correlated with the consumption of FLVs, and negatively correlated with the ingestion of fats. 9

Evidence suggests that the DTAC is directly associated with a lower risk of all-cause mortality, cancer, and cardiovascular disease. 10 In a study conducted among pregnant women, it was found that women classified in the third tertile of the DTAC estimation had a 46% lower chance of premature births, regardless of confounding factors. In addition, at an intermediate level, the DTAC was associated with a 75% lower chance of LBW infants. 11

Sociodemographic and lifestyle characteristics and the presence of morbidities influence food choices. Studies suggest that older pregnant women, who live with a partner, who perform paid work and have a higher level of education tend to adopt more diverse and healthier diets. 12 Conversely, it has been observed that younger pregnant women present greater adherence to the patterns of snacks (breads, cheese, sweets, and chocolate, among others). 13 However, we are unaware of the existence of studies that have investigated the factors associated with higher DTAC estimates in pregnant women.

The aim of the present study was to investigate the factors associated with higher DTAC estimates in pregnant women and to identify the main dietary sources of the DTAC.

## Methods

This is a secondary analysis of a cross-sectional study conducted with 785 adult pregnant women attended at Primary Health Units of the Brazilian National Health System (SUS, in the Portuguese acronym) in the city of Ribeirão Preto, state of São Paulo, Brazil. Conducted between 2011 and 2012, the study aimed to investigate the association between the usual diet during pregnancy and GDM, as described in detail in the publication by Barbieiri et al. 14

The pregnant women were invited to participate in the study when the oral glucose tolerance test (OGTT) was performed. A shift plan was established in five laboratories with the health department of the municipality. All interviews were conducted by previously trained nutritionists. The women that fulfilled the inclusion criteria and agreed to participate in the study were interviewed after signing the consent form.

Fasting blood samples, 1 and 2 hours after ingestion of a 75 g glucose overload, were obtained from all study participants. The glucose oxidase method was used to determine plasma glucose. The diagnosis of GDM was based on the 2014 World Health Organization (WHO) criteria. 15

The sample size calculation was based on the primary outcome of the study, GDM. Considering a prevalence of 20% of GDM among adult women attended in the SUS, with an acceptable margin of error of 5%, a sample of 512 pregnant women was necessary. 16 Considering that, in logistic regression models, 10 cases are required for each exploratory variable, the sample was considered sufficient for the analysis of the present study. 17

**Fig. 1:**
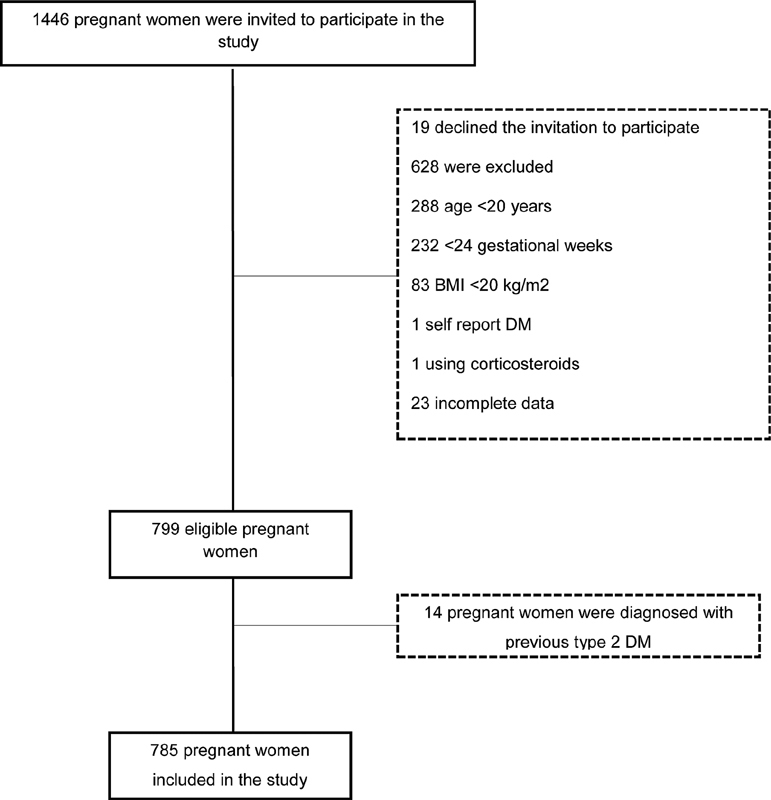
Flowchart of the study.

Women aged ≥ 20 years, with pregestational BMI ≥ 20kg/m ^2^ and screened for GDM from the 24 ^th^ gestational week were included. Pregnant women diagnosed with previous type 1 or type 2 diabetes mellitus (DM), twin pregnancies, who reported use of drugs that altered blood glucose (such as glucocorticoids) and reported diseases that interfered with their habitual food consumption (chronic renal failure, acquired immunodeficiency syndrome or cancer) were excluded. In total, 1,446 women were invited to participate in the study, with 785 eventually included, as described in Fig. 1 .

The estimation of the diet of the pregnant women was performed by means of 2 24-hour dietary recalls (24hR), following the “multiple-pass” methodology in 3 stages, between the 24 ^th^ and 39 ^th^ gestational weeks. The first 24hR was collected from all pregnant women on the day of the face-to-face interview. 18 The second 24hR was obtained from a subsample of 73% of the pregnant women through telephone contact, on nonconsecutive days, with at least 1 week between replications, regardless of the day of the week or season.

For the estimation of dietary nutrients, the Brazilian Table of Chemical Composition of Food (TACO) was used with the NutWin® Program (Programa de Apoio à Nutrição. Version 1.5. São Paulo: Escola Paulista de Medicina; 2002). 19 The underreporting of energy intake was estimated using the method by Goldberg et al. 20 , adopting a cutoff point of 1.35 for the ratio between the energy estimate and the basal metabolic rate.

The DTAC (in mmol/100 g), represented by the sum of the TAC of the food and the TAC determined in the supplements, was estimated using data from previous studies that established the total amount of antioxidant present in food and drink through the Ferric Reducing Antioxidant Power assay (FRAP). 8 21 For foods without TAC determination, the value of a similar food item or of the same botanical group was used: for chicory, endive and arugula, the spinach estimate was used. For red fruits, nectarines and cream, the TAC values of strawberry, peach and butter were used, respectively. For cooked foods that did not have certain TAC values, the value of the fresh food was used. To obtain the TAC value of homemade preparations, previously broken down into ingredients, the calculation of the TAC of each item of the recipe was performed. It was not possible to consider the TAC values of coconut water, salted cod, cane juice, flaxseed, maxixe, sardines, sova, and sushi, as the TAC has not been established for these foods.

The TAC of dietary supplements was estimated by multiplying the amount of antioxidant compound (mg or IU) present in the capsule by its TAC value (mmol/100 g). Of the antioxidant compounds present in the dietary supplements, it was not possible to estimate the TAC values of thiamine (B1), riboflavin (B2), nicotinamide (B3), pantothenic acid (B5), and biotin (B7), as the TAC has not been determined for these.

To estimate the usual DTAC, the Multiple Source Method (MSM) was used. This program was developed by the European Prospective Investigation in Cancer and Nutrition (EPIC) ( https://msm.dife.de/ ). 22 The MSM estimates the usual intake of food and nutrients through the product of the probability of intake and the usual intake, corrected for variability. Correction for variability in intake eliminates the need for a large number of 24hR replications. The DTAC estimate was adjusted for the total dietary calories using the residual method. 23

Information on age, self-reported skin color, education of the pregnant woman and of the head of the family, possession of items, occupation, marital status, parity, physical activity, smoking, consumption of alcoholic beverages, and use of dietary supplements was obtained by through a structured questionnaire. For the classification of the economic stratum, the Brazil Economic Classification Criterion was used, which defines the strata from A (highest level) to E (lowest level), based on the possession of items and on the educational level of the head of the family. 24

Weight (kg) and height (m) measurements were obtained using a digital scale (TANITA model HS302 Arlington Heights, Illinois, 60005, USA) and a portable stadiometer (SANNY model ES2040 São Bernardo do Campo, São Paulo, 09628-060, Brazil), respectively. Gestational age was estimated from the date of the last menstrual period recorded on the card of the pregnant woman and was subsequently corrected through ultrasound. Pregestational weight was self-reported and corrected from recording on the card of the pregnant woman. The Institute of Medicine criteria were used to assess the adequacy of pregestational BMI (kg/m ^2^ ). 25

Descriptive data were presented as mean ± standard deviation (SD) or median (P25, P75) for continuous variables, and *n* (%) for categorical variables. To investigate differences in maternal characteristics according to the DTAC tertiles, the chi-squared test ( *X* ^2^ ) was used for the categorical variables and analysis of variance (ANOVA) or the Kruskal-Wallis tests for the continuous variables. The Spearman coefficient was used to investigate the correlation between the DTAC and the food and nutrient groups of the maternal diet.

The factors associated with the higher DTAC estimate (≥ 4.3 mmol/day) were investigated in age-adjusted backward logistic regression models (20–25/25–30/30–35/≥ 35 years old), self-reported skin color (white/not white), socioeconomic strata (A + B/C/D + E), living with a partner (yes/no), paid work (yes/no), pregestational BMI (kg/m2), current smoker (no/yes), physical activity (< 150 minutes/≥ 150 minutes of walking and/or physical exercises per week), education (< 4/4–8/≥ 9 years of study), parity (< 1/≥ 2 children), consumption of alcoholic beverages (yes/no), use of food supplement (yes/no), underreport of energy intake (yes/no), total dietary energy (kcal/day tertiles), and gestational trimester at the time of the interview (2 ^nd^ /3 ^rd^ ). The associated factors investigated were determined through theoretical assumptions; however, the final models were established based on backward selection.

*P* values <.05 were considered significant. Statistical analyses were performed using the SPSS Statistics for Windows, version 17.0 (SPSS Inc., Chicago, IL, USA).

The present article complies with the ethical principles contained in the Declaration of Helsinki. The study was approved by the Research Ethics Committee of the School Health Center of the Faculdade de Medicina de Ribeirão Preto (Auth. No. 014/2018-CEP/CSE - FMRP - USP).

## Results

In total, 785 pregnant women were investigated. The mean (SD) age of the pregnant women was 28 (5) years old, and the education level varied between zero and 15 years of study. Among them, 25% were overweight and 32% were obese. The mean (SD) of the DTAC exclusively from the diet was 4.7 (2.4) mmol/day, with the mean considering the use of dietary supplements being 4.8 (2.5) mmol/day. The median (P25, P75) of the DTAC was 4.3 (3.3–5.6) mmol/day. Among the women classified in the third tertile of the DTAC, a greater proportion of self-reported white skin color presented a lower mean pregestational BMI when compared with the pregnant women with lower DTAC estimates ( Table 1 ).

**Table 1:** Sociodemographic and lifestyle characteristics according to tertiles of the estimated dietary total antioxidant capacity of the pregnant women ( *n* = 785)

	DTAC tertiles a			*p-value* b
	T1 ( *n* = 261)	T2 ( *n* = 262)	T3 ( *n* = 262)	
Age (years old)	27 ± 5.2	28 ± 5.7	27.6 ± 5.51	0.07
White skin color self-reported	119 (45.6)	102 (38.9)	131 (50.0)	0.04
Education (years of study)				
< 4	8 (3.1)	9 (3.4)	10 (3.8)	0.13
4 to 8	67 (25.7)	84 (35.9)	94 (32.1)	
≥ 9	186 (71.3)	159 (60.7)	168 (64.1)	
Paid work	113 (43.3)	117 (44.8)	134 (51.1)	0.16
Socioeconomic stratum				
A + B	59 (22.6)	45 (17.2)	50 (19.1)	0.29
C	175 (67.0)	179 (68.3)	172 (65.6)	
D + E	27 (10.3)	38 (14.5)	40 (15.3)	
Living with partner	209 (80.1)	201 (76.7)	206 (78.6)	0.64
Parity (number of children)	1.11 ± 1.21	1.2 ± 1.29	1.2 ± 1.19	0.37
Pre-gestational body mass index (kg/m ^2^ )	26.44 ± 5.6	25.86 ± 5.6	25.27 ± 4.5	0.02
Practice physical activity c	30 (0.0; 120.0)	45 (0.0; 140.0)	50 (0.0; 142.5)	0.14
Current smoker	24 (9.2)	22 (8.4)	25 (9.5)	0.44
Consumption of alcoholic beverages	68 (26.1)	70 (26.7)	59 (22.5)	0.50
Use of food supplement	153 (58.6)	170 (65.1)	176 (67.2)	0.10
Energy (kcal/day)	1981.55 ± 554.60	1956.60 ± 542.23	1926.25 ± 538.45	0.51
Energy underreport	136 (52.1)	109 (41.6)	124 (47.0)	0.06
Gestational trimester at interview				
Second	130 (49.8)	147 (56.1)	139 (53.1)	0.35
Third	131 (50.2)	115 (43.9)	123 (46.9)	

Values presented as mean ± SD or *n* (%) or median (P25; P75).

a DTAC, Dietary total antioxidant capacity. For the analysis, the DTAC adjusted for the total dietary calories, using the residual method, was considered. T, tertile. TAC (mmol/day) Mean (SD) minimum- maximum: T1 (2.8 [0.62], 0.3–3.6); T2 (4.4 [0.4], 3.6–5.2); T3 (6.9 [2.0], 5.2–21.84).

b *p* <.05 according to ANOVA test for the continuous variables with normal distribution, Kruskal-Wallis test for the continuous variables without normal distribution, and chi-squared test for the categorical variables.

c Minutes of walking or exercise/week

A positive correlation was observed between the DTAC and the estimates of carbohydrates, fiber, vitamin A, vitamin E, folic acid, and the consumption of sugar, coffee and tea, beans, fruits, vegetables, dairy products, breads, and natural fruit juice. Conversely, there was a negative correlation between the DTAC and the consumption of snacks and sandwiches, soft drinks, and artificial juices ( Table 2 ).

**Table 2:** Characteristics of the diet according to the tertiles of the estimated dietary total antioxidant capacity of pregnant women ( *n* = 785)

	DTAC tertiles a			
	Spearman correlation. ( *r* )	T1 ( *n* = 261)	T2 ( *n* = 262)	T3 ( *n* = 262)
Nutrients				
Carbohydrate (%TEV)	0.19 c	53 (49; 58)	54 (51; 58)	56 (52; 59)
Protein (%TEV)	0.01	16 (14; 19)	16 (14; 18)	16 (14; 18)
Lipid (%TEV)	−0.05	24 (21; 28)	24 (21; 27)	24 (21; 27)
Fiber/1000kcal	0.22 c	11 (8; 13)	11 (9; 14)	12 (10; 14)
Vitamin A (mg)	0.14 c	305 (219; 393)	346 (265; 468)	345 (262; 464) b
Vitamin B12 (mg)	0.06	4 (3; 6)	5 (3; 7)	5 (3; 6)
Vitamin C (mg)	0.32	30 (13; 89)	60 (22; 137)	102 (35; 197) b
Vitamin E (mg)	0.18 c	4 (3; 5)	4 (3; 5)	4 (4; 6) b
Iron (mg)	0.05	63 (8; 69)	65 (9; 69)	65 (9; 69)
Zinc (mg)	0.00	10 (8; 12)	10 (8; 12)	10 (8; 12)
Selenium (mg)	−0.23	82 (69; 95)	80 (69; 95)	77 (67; 99)
Folic Acid (µg)	0.23 c	365 (302; 459)	399 (333; 490)	429 (344; 537) b
Foods				
Chocolate (g)	−0.02	2 (1; 10)	2 (1; 21)	1 (1; 11) b
Sugar (g)	0.37 c	2 (1; 5)	5 (2; 10)	9 (4;14) b
Rice (g)	−0.04	218 (150; 301)	215 (159; 277)	201 (158; 267)
Coffee/Tea (ml)	0.49 c	8 (4; 28)	29 (6; 666)	74 (28; 111) b
Beans (g)	0.09 c	88 (52; 149)	110 (59; 149)	102 (71; 149)
Fruits (g)	0.31 c	53 (31; 116)	87 (35; 160)	132 (57; 216) b
Snacks and sandwiches (g)	−0.13 c	11 (7; 42)	9 (6; 25)	9 (6; 13) b
Legumes (g)	0.16 c	39 (24; 66)	47 (23; 78)	57 (30; 85)
Milk/Yogurt (ml)	0.28 c	133 (38; 240)	199 (97; 277)	221 (133; 304) b
Eggs (g)	0.02	2 (2; 3)	2 (2; 3)	2 (2; 3)
Bread (g)	0.11 c	49 (18; 55)	54 (31; 68)	54 (29; 68) b
Soda/Artificial Juice (ml)	−0.21 c	487 (230; 712)	357 (193; 580)	314 (154; 535) b
Natural Juice (ml)	0.16 c	3 (2; 3)	3 (3; 2)	3 (2; 171) b
Root vegetables (g)	0.01	18 (11; 27)	17 (9; 39)	18 (10; 28)

a DTAC, Dietary total antioxidant capacity. For the analysis, the DTAC adjusted for the total dietary calories, using the residual method, was considered. TAC (mmol/day) Mean (SD) minimum - maximum: T1 (2.8 [0.62], 0.3–3.6); T2 (4.4 [0.4], 3.6–5.2); T3 (6.9 [2.0], 5.2–21.84).

b *p* <.05 according to the Kruskal-Wallis test (continuous variables without normal distribution).

c *p* <.05 according to Spearman Correlation test ( *r* )

**Fig. 2:**
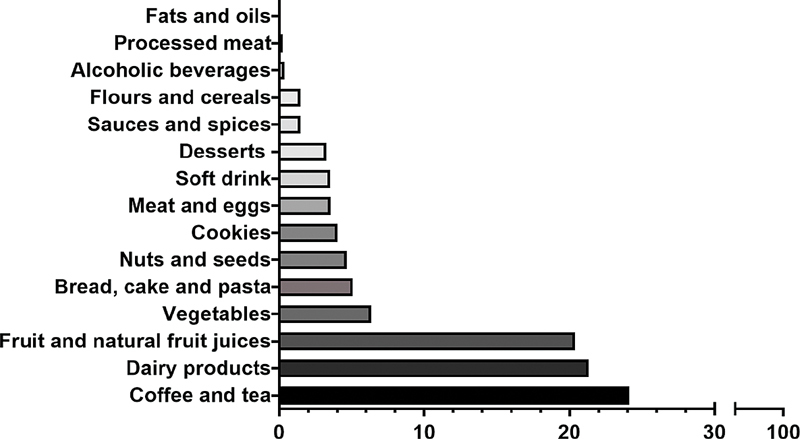
Main dietary contributors to the DTAC intake of the pregnant women ( *n* = 785).

The main food groups that contributed to the DTAC estimate were coffee and tea (24.12%), dairy products (21.34%), fruits, and natural fruit juices (20.37%) ( Fig. 2 ).

In the backward logistic regression models, it was found that pregnant women ≥ 35 years old were twice as likely to have an antioxidant intake above the median when compared with the younger pregnant women. The women that were overweight or obese in the pregestational period had a 37 and 41% lower likelihood of the DTAC estimate being above the median, respectively, when compared with the eutrophic pregnant women. Those that reported using dietary supplements during pregnancy had a 39% higher chance of ingesting DTAC above the median when compared with those who did not ingest them. There was also an inverse relationship between total dietary energy intake and the higher DTAC estimate. There was no significant association between the DTAC and the other factors investigated ( Table 3 ).

**Table 3:** Factors associated with the determination of the DTAC above the median (≥ 4.3 mmol/day) ( *n* = 785)

	Total Antioxidant Capacity a					
	Unadjusted model			Adjusted model b		
	OR	95%CI	*p-* value	OR	95%CI1	*p-value*
Age (years old)			.03			0.02
20–25	1	−		1	−	
25–30	1.20	0.85–1.69		1.21	0.85–1.72	
30–35	1.12	0.75–1.65		1.16	0.77–1.73	
≥ 35	1.89	1.18–3.03		2.01	1.24–3.27	
Skin color self-reported			.24			
White	1	−				
Non-white	0.84	0.64–1.12				
Socioeconomic stratum			.11			
A + B	1	−				
C	1.06	0.74–1.52				
D + E	1.56	0.95–2.57				
Marital status			.61			
Married	1	−				
Single, widowed or separated	0.91	0.65–1.28				
Working			.61			
No	1	−				
Yes	1.08	0.81–1.42				
Pregestational body mass index			.01			0.003
< 25	1	−		1	−	
25–29	0.66	0.47–0.91		0.63	0.45–0.89	
≥ 30	0.66	0.45–0.98		0.59	0.40–0.88	
Current smoker			.89			
No	1	−				
Yes	1.03	0.63–1.68				
Practice physical activity			.73			
< 150 minutes	1	−				
≥ 150 minutes	1.06	0.76–1.49				
Education (years of study)			.13			
< 4	1	−				
4 to 8	0.86	0.39–1.91				
≥ 9	0.70	0.32–1.52				
Parity			.86			
≤ 1	1	−				
≥ 2	1.03	0.76–1.39				
Consumption of alcoholic beverages			.55			
No	1	−				
Yes	0.91	0.65–1.25				
Use of food supplement			.02			0.03
No	1	−		1	−	
Yes	0.71	0.53–0.95		1.39	1.03–1.88	
Energy underreport			.94			
No	1	−				
Yes	0.98	0.74–1.31				
Total dietary energy (kcal/d) c			.01			
T1	1	−		1	−	0.004
T2	0.64	0.46–0.91		0.62	0.44–0.89	
T3	0.64	0.45–0.90		0.59	0.42–0.85	
Gestational trimester at the interview			.56			
2 ^nd^	1	−				
3 ^rd^	0.92	0.69–1.22				

a DTAC Adjusted for total dietary calories, using the residual method. Median DTAC 4.3 mmol/day.

b Logistic regression model with age-adjusted backward function (20–25/25–30/30–35/≥ 35 years old), self-reported skin color (white/non-white), socioeconomic strata (A + B/C/D + E), living with partner (yes/no), paid work (yes/no), pre-pregnancy body mass index (kg/m ^2^ ), current smoker (no/yes), physical activity (< 150 minutes/≥ 150 minutes of walking and/or physical exercises/week), education (< 4/4–8/≥ 9 years of study), parity (< 1/≥ 2 children), consumption of alcoholic beverages (yes/no), use of food supplement (yes/no), underreport of energy intake (yes/no) total dietary energy (kcal/day tertiles) and gestational trimester at the time of the interview (second/third).

c Total dietary energy (kcal/d): T1: 644–1695 kcal/d; T2: 1696–2129 kcal/d; T3: 2130–4464 kcal/d.

## Discussion

Among the pregnant women investigated, women ≥ 35 years old, with pregestational BMI < 25kg/m ^2^ , and who reported using dietary supplements during pregnancy had a greater chance of ingesting antioxidants above the median DTAC (≥ 4.3mmol/day). However, it was observed that pregnant women with higher energy intakes had less chance of ingesting antioxidants above the median DTAC (≥ 4.3mmol/day).

The median DTAC observed among the pregnant women using the FRAP method was 4.3mmol/day, which is much lower than that reported in studies with adults in Spain (17mmoL/day) and France (13mmoL/day). 26 27 The findings of the present study agree with previous evidence that suggests a high prevalence of inadequate intake of nutrients with antioxidant properties by pregnant women in Brazil. 28 The discrepancy in DTAC values observed in the present study, when compared with data from other countries, can be partially explained by the low consumption of fruits and vegetables by the pregnant women in the sample. The mean daily consumption of fruits and vegetables observed was 87.30 g and 43.37 g, respectively, values well below those recommended by the WHO. 29

In the present study, the coffee and tea groups (24.12%), dairy products (21.34%), and fruits and natural fruit juices (20.37%) were the ones that most contributed to the DTAC estimate of the usual diet of the pregnant women. Coffee stood out as the main DTAC source both due to its high antioxidant capacity and its high consumption, corroborating findings of other studies. 13 30 However, coffee consumption during pregnancy should be moderate because caffeine is absorbed rapidly upon ingestion and passes the placental barrier leading to higher exposure for the fetus. 30 Observational studies suggest that excess intake of caffeine may be associated with negative birth results. In a meta-analysis of observational studies, there was a 3% increase in the risk of babies with low birth weight (LBW) for each additional 100 mg of caffeine consumed per day during pregnancy. 31

The milk and dairy products group was the second group that most contributed to the determination of the DTAC of pregnant women. Despite the fact that milk is not a relevant source of TAC, a high consumption was verified in the study population. The group of fruits and natural fruit juices was the third that most contributed to the determination of DTAC. However, the mean consumption of fruit by the pregnant women was below that recommended by the WHO, contributing to a low mean intake of antioxidants. 29

There was a positive correlation between sugar consumption and the DTAC. A possible explanation for this unexpected result is that sugar is commonly added to some of the DTAC source foods by the study population, such as coffee, tea, milk, and natural fruit juices.

The DTAC, in addition to measuring antioxidant intake, can be considered a potential marker of diet quality, allowing a comparison between our results and studies that assessed diet quality using dietary indices. 10 The data from the present study suggest a higher chance of estimating the DTAC above the median among eutrophic women, in agreement with previous evidence. In a study conducted by Laraia et al., 32 the pregestational BMI was inversely associated with the quality of the diet of pregnant women, evaluated using the Diet Quality Index for Pregnancy (DQI-P).

A direct relationship between the age of the pregnant women and the DTAC was observed in the present study, corroborating studies that also found better quality diets in older pregnant women. 13 33 One of these studies, conducted with Brazilian pregnant women, found that older women presented greater adherence to a “healthy” dietary pattern, composed of vegetables and legumes, fruits, and natural fruit juices. 14

The mean (SD) of the TAC exclusively from the diet was 4.7 (2.4) mmol/day, with the mean considering the use of dietary supplements being 4.8 (2.5) mmol/day. There was a small variation in the DTAC when considering the use of a dietary supplement; however, this showed a direct relationship with the higher median of the DTAC. The combination of dietary sources rich in antioxidants and the use of supplements contributes to favorable health outcomes for the baby. However, further studies in other populations are needed. Pregnant women with lower energy intake were more likely to present DTAC estimates above the median. Evidence show that healthier diets are rich in foods with a high nutrient density and low energy density, as they are based on foods of plant origin. Consequently, they are rich in vitamins and minerals with antioxidant properties, as well as rich in fiber. 34

The present study has some limitations, the main one being its cross-sectional design. The DTAC determination of the diet of the pregnant women was based mainly on an international database, in which the values may vary in relation to the food produced in Brazil, considering the differences in soil and climate. The underreporting of energy intake was estimated using the formula by Goldberg et al. 20 , adopting a cutoff point ≤ 1.35. 20 The high proportion of underreporting of the diet among the participants in our study (47%) corroborates an investigation conducted among pregnant women in Ireland, in which the underreporting of energy intake was 42%, even adopting a cutoff ≤ 1.2. 35 It is important to note that the method by Goldberg et al. 20 may not be the best approach to estimate the underreporting of the energy intake during pregnancy, as it does not consider the practice of physical activity of the individual and presupposes the maintenance of body weight. In addition, the cutoff point of the BMI adopted as an inclusion criterion in the study was ≥ 20kg/m ^2^ , not allowing the extrapolation of data for pregnant women with lower BMI values.

Among the strengths of the present study, its originality stands out. Furthermore, the data collection was performed by trained nutritionists, and the DTAC estimate was performed using 24hR adjusted through the MSM. Future studies are necessary to confirm the findings of the present study.

## Conclusion

In conclusion, pregnant women ≥ 35 years old, eutrophic, using dietary supplements, and with lower energy intake had a greater likelihood of presenting DTAC estimates above the median. It should be highlighted that the DTAC of the evaluated pregnant women was the lowest value ever described in the literature, showing the importance of developing public policies that encourage adherence to the recommendations for the consumption of FLVs during the different life cycles.
